# Molecular Characterization of a Novel *Enamovirus* Infecting Raspberry

**DOI:** 10.3390/v15122281

**Published:** 2023-11-21

**Authors:** Igor Koloniuk, Jana Fránová, Jaroslava Přibylová, Tatiana Sarkisova, Josef Špak, Jiunn Luh Tan, Rostislav Zemek, Radek Čmejla, Martina Rejlová, Lucie Valentová, Jiří Sedlák, Jan Holub, Jan Skalík, Dag-Ragnar Blystad, Bijaya Sapkota, Zhibo Hamborg

**Affiliations:** 1 Institute of Plant Molecular Biology, Biology Centre, Czech Academy of Sciences, 370 05 Ceske Budejovice, Czech Republic; jana@umbr.cas.cz (J.F.); pribyl@umbr.cas.cz (J.P.); sarkisova@umbr.cas.cz (T.S.); spak@umbr.cas.cz (J.Š.); 2 Institute of Entomology, Biology Centre, Czech Academy of Sciences, 370 05 Ceske Budejovice, Czech Republic; jiunnluh@gmail.com (J.L.T.); rosta@entu.cas.cz (R.Z.); 3 Faculty of Science, University of South Bohemia, 370 05 Ceske Budejovice, Czech Republic; 4 Research and Breeding Institute of Pomology Holovousy Ltd., 508 01 Horice, Czech Republic; radek.cmejla@vsuo.cz (R.Č.); martina.rejlova@vsuo.cz (M.R.); lucie.valentova@vsuo.cz (L.V.); jiri.sedlak@vsuo.cz (J.S.); 5 Jan Holub Ltd., 783 25 Bouzov, Czech Republic; info@janholub.cz (J.H.); lab@janholub.cz (J.S.); 6 Norwegian Institute of Bioeconomy Research, 1433 Aas, Norway; dag-ragnar.blystad@nibio.no (D.-R.B.); bijaya.sapkota@nibio.no (B.S.); zhibo.hamborg@nibio.no (Z.H.)

**Keywords:** raspberry, HTS, Rubus, virus, aphid transmission

## Abstract

Raspberry plants, valued for their fruits, are vulnerable to a range of viruses that adversely affect their yield and quality. Utilizing high-throughput sequencing (HTS), we identified a novel virus, tentatively named raspberry enamovirus 1 (RaEV1), in three distinct raspberry plants. This study provides a comprehensive characterization of RaEV1, focusing on its genomic structure, phylogeny, and possible transmission routes. Analysis of nearly complete genomes from 14 RaEV1 isolates highlighted regions of variance, particularly marked by indel events. The evidence from phylogenetic and sequence analyses supports the classification of RaEV1 as a distinct species within the *Enamovirus* genus. Among the 289 plant and 168 invertebrate samples analyzed, RaEV1 was detected in 10.4% and 0.4%, respectively. Most detections occurred in plants that were also infected with other common raspberry viruses. The virus was present in both commercial and wild raspberries, indicating the potential of wild plants to act as viral reservoirs. Experiments involving aphids as potential vectors demonstrated their ability to acquire RaEV1 but not to successfully transmit it to plants.

## 1. Introduction

Raspberry plants (*Rubus* spp.) are susceptible to a variety of viruses that can significantly impact their crop yield and quality [1]. Recently, with the advancement and cost reduction of high-throughput sequencing (HTS), many new viruses have been unveiled that infect a range of plant species, including *Rubus* species [2,3]. In the age of modern genomic research, our understanding of plant pathogens has undergone a transformative shift. However, it is paramount to highlight that many of these newly discovered viruses have been only partially characterized using sequence data, leaving gaps in our comprehensive understanding of their biology, epidemiology, and potential impact.

The vegetative propagation of raspberry plants increases the likelihood of rapid viral spread. However, *Rubus*-infecting viruses belong to different taxa and employ a range of transmission modes [3,4,5]. Beyond vegetatively propagated plant material, they can also spread via invertebrate vectors, seeds, pollen, or a combination of these routes [4,6]. Different aphid species have been reported to transmit viruses with varying efficiencies, and these interactions are often shaped by highly specific relationships [7,8].

Enamoviruses, similar to other viruses in the family *Solemoviridae,* are transmitted by sap-feeding aphid vectors [9]. They circulate within the vector, interacting with proteins in its gut and accessory salivary glands before transmission to a new plant. The aphid’s gut therefore serves as the initial transmission barrier, ensuring selectivity in virus uptake [10,11]. Importantly, virus replication occurs only within the plant phloem [12,13]. Viral particles consist of single-stranded RNA and viral proteins, mainly coat protein (CP) and a smaller amount of CP fused with the P5 readthrough product, known as the CP-readthrough domain (CP-RTD) [11]. The translation of the CP-RTD takes place from subgenomic RNA (sgRNA) and is governed by a complex of events and regulated by downstream distal and proximal RNA regulatory sequences and structures [14]. The involvement of the sgRNA strategy in the replication cycle of enamoviruses as well as other luteoviruses is thought to facilitate recombination events during replicase strand switching at sgRNA promoters [15].

In this study, we characterize a novel virus, provisionally named raspberry enamovirus 1 (RaEV1). Genomic sequences from fourteen distinct RaEV1 isolates from the Czech Republic and Norway were obtained. Phylogenetically, RaEV1 is closely related to members of the *Enamovirus* genus. The virus’s incidence was studied in wild and cultivated raspberry plants, and we probed its presence within aphid colonies associated with the chosen samples. Furthermore, our study examined the coinfection frequency with other raspberry viruses, some of which are being reported in the Czech Republic and Norway for the first time, namely black raspberry necrosis virus (BRNV), raspberry leaf blotch virus (RLBV), raspberry leaf mottle virus (RLMV), raspberry vein chlorosis virus (RVCV), and Rubus yellow net virus (RYNV). Transmission experiments involving three aphid species were also conducted.

## 2. Materials and Methods

### 2.1. Plant Materials

A *Rubus idaeus* plant (cultivated under tissue culture and as a mother plant growing in the field), isolate GR (cultivar Ispolin/Gordost Rosyi), was provided by Jan Holub Ltd. (Bouzov, Czech Republic). For virus screening, *R. idaeus* plant samples were collected from various locations throughout the Czech Republic. These samples included plant material from germplasm collection, commercial plantings, gardens, CAC-certified propagation plants, and hobby markets. When possible, colonizing arthropods, mainly aphids, were collected in parallel.

Two *R. idaeus* plants (one wild raspberry, named ALOJ-12, and one cultivated raspberry of cv. Glen Ampel, named ALOJ-13) were provided by a raspberry grower in County Agder, Norway.

For virus transmission with aphids, RaEV1-infected raspberry shoots from ALOJ-13 and GR raspberry plants were used as RaEV1 source material. Therefore, the roots of raspberry samples were collected and grown in a quarantine greenhouse under 16 °C/10 °C and a 16/8 h light cycle.

Virus-free raspberry plants rooted from certified raspberry tissue culture plants, provided by the Norwegian company Sagaplant AS (Akkerhaugen, Norway), and *Chenopodium quinoa* plants grown from seeds were used as plants for inoculation.

### 2.2. RNA Isolation and cDNA Synthesis

For samples of Czech origin, total RNA was isolated from approximately 100 mg of fresh or frozen (−20 °C) young raspberry plant leaves, from the whole bodies of aphids and small invertebrates, or from the heads and thoraxes of insects using a Ribospin Plant Kit (GeneAll Biotechnology, Seoul, Republic of Korea), following the manufacturer’s instructions. Reverse transcription was carried out using M-MLV Reverse Transcriptase (Invitrogen, Waltham, MA, USA), and 0.5 µg of RNA in the case of plants or less in the case of arthropods was used as a template for each 20 µL reaction.

For the samples of Norwegian origin, plant RNA extractions were carried out using a Norgen Plant/Fungi RNA Kit (Norgen Biotek Corp, Thorold, ON, Canada) according to the manufacturer’s instructions, with some modifications. The quantity of RNA was assessed using a NanoDrop 1000b spectrophotometer (NanoDrop Technologies, Wilmington, DE, USA), and the extracted RNA was stored at a temperature of −80 °C for future use. The aphid samples were collected with DNA/RNA Shield Reagent Products (Zymo Research, Irvine, CA, USA) and stored at −80 °C. Frozen samples were crushed directly using a small glass rod and TRIzol reagent (600 μL) (Thermo Fisher Scientific, Waltham, MA, USA). RNA from aphids were extracted via a Direct-zol RNA Miniprep Kit (Zymo Research), according to the manufacturer’s instructions. The purified RNA was then stored at −80 °C. Complementary DNA synthesis was performed using SuperScript IV Reverse Transcriptase (Invitrogen) with 1 µg of RNA as a template.

### 2.3. RACE (Rapid Amplification of cDNA Ends)

Viral genomic termini were determined using 5′- and 3′-RACE kits (Invitrogen) with virus-specific primers (Table S1). Prior to the 3′ terminus determination, the total RNA was first polyadenylated with poly(U) polymerase and adenosine triphosphate (NEB, Ipswich, MA, USA), following the manufacturer’s recommendations. The resulting RACE products were then purified and subjected to direct sequencing (Eurofins Genomics, Luxembourg).

### 2.4. RT-PCR and Sanger Sequencing

The second step of the reverse transcription PCR was performed using the above-described cDNA preparation step. One microliter of each cDNA preparation was mixed with 10 µL of 2× PPP Master Mix (Top-Bio, Vestec, Czech Republic), 8 µL of PCR-grade H_2_O, and the corresponding primers at 0.5 µM (Table S1). Reaction mixtures devoid of cDNA templates served as no-template controls. The RNA isolated from the lamina tissue of the raspberry cv. GR where RaEV1 was previously identified via HTS (no. B298, isolate GR) was employed as a positive control in all PCR assays. For molecular identification of arthropods, cDNA was amplified through PCR with primers specific to the cytochrome C oxidase subunit (COI), as previously described [16]. Each PCR product (4 µL) was analyzed via electrophoresis in a 1% agarose gel prestained with GelRed (Biotium, Hayward, CA, USA). DNA bands were visualized using a UV transilluminator.

For the sample of Norwegian origin, PCR was carried out in a 25 µL reaction with Taq DNA Polymerase (Thermo Fisher Scientific), following the manufacturer’s recommendation, with 2 µL of cDNA.

For Sanger sequencing, the bands were either excised from the 1.5% agarose gel or purified directly from the PCR mixture using the Expand Combo Mini Kit (GeneAll). The purified PCR products were sequenced in both directions (Eurofins Genomics, Luxembourg). All sequences were identified using the BLAST database provided by the NCBI.

### 2.5. RT-qPCR

Reverse transcription qPCR was performed using qPCR 2× Blue Master Mix (Top-Bio, Czech Republic), the primers RaspEnaV-F02 and RaspEnaV-FR02, and the probe RaspEnaV-Pr01 (Table S1) at final concentrations of 500 nM, 500 nM, and 200 nM, respectively. Two microliters of freshly prepared undiluted cDNA was used as a template. PCR was run on a Rotor-Gene Q Cycler (Qiagen, Hilden, Germany) with the following parameters: 95 °C/5 min; 50 cycles 95 °C/20 s, 58 °C/20 s, and 72 °C/20 s. The results were analyzed using Rotor-Gene Q bundled software (Version 2.3.4).

### 2.6. High-Throughput Sequencing and Viral Genome Sequencing

The HTS sequencing library for the GR isolate was prepared from enriched double-stranded RNA [17] using the NEBNext Ultra II Directional RNA Library Prep Kit for Illumina (NEB) and then processed on the Illumina NovaSeq-S4 platform in PE 150 output mode (Admera Health Biopharma Services, South Plainfield, NJ, USA).

For ALOJ-12 and ALOJ-13 samples, total RNA was isolated and on-column DNase treatment was applied during RNA extraction for the samples applied to HTS. RNA quality was assessed via an Agilent 2100 Bioanalyzer (Agilent Technologies, Santa Clara, CA, USA). RNA samples with RIN (RNA integrity number) scores greater than 5.0 were used for HTS. The RNA library was prepared, and the sequencing was carried out using a paired-end (2 × 150) configuration on the NovaSeq 6000 platform (Fasteris, Life Science Genesupport SA, Plan-Les-Ouates, Switzerland).

Validation of the HTS data was performed via RT-PCR and the Sanger sequencing of the obtained products.

Based on the viral genomic sequence from the GR isolate, primers were designed to cover the whole viral genome in 9 overlapping fragments to obtain the complete protein-coding genomic sequences for other isolates (Table S1). The amplicons were analyzed via electrophoresis in a 1% agarose gel, and the fragments were excised, purified, and Sanger-sequenced.

### 2.7. Aphid Cultures

Virus-free colonies of *Aphis idaei*, *Amphorophora rubi idaei*, and *Myzus persicae* were established as individual lines from single eggs. *A. idaei* and *Am. rubi idaei* were reared on virus-free raspberry rooted from tissue culture plants and *M. persicae* was reared on virus-free pepper plants grown from seeds. The aphids were kept in different aphid mesh chambers in an aphid culture room with 18 °C, 75% humidity under a 16 h light/8 h dark cycle. All experiments were conducted under separate aphid mesh chambers under the same conditions.

### 2.8. Virus Transmission Experiment

Tested RaEV1-free colonies of *A. idaei*, *Am. rubi idaei*, and *M. persicae* were used. The starvation time for different aphids was determined by not feeding the aphids for different periods for up to two hours and then offering them raspberry leaves to observe whether the aphids fed instantly or not. The feeding process was observed through a stereo microscope (Lecia MZ72). A starvation time of one hour and wingless adults were used before virus acquisition treatments in all experiments.

**Figure 1:**
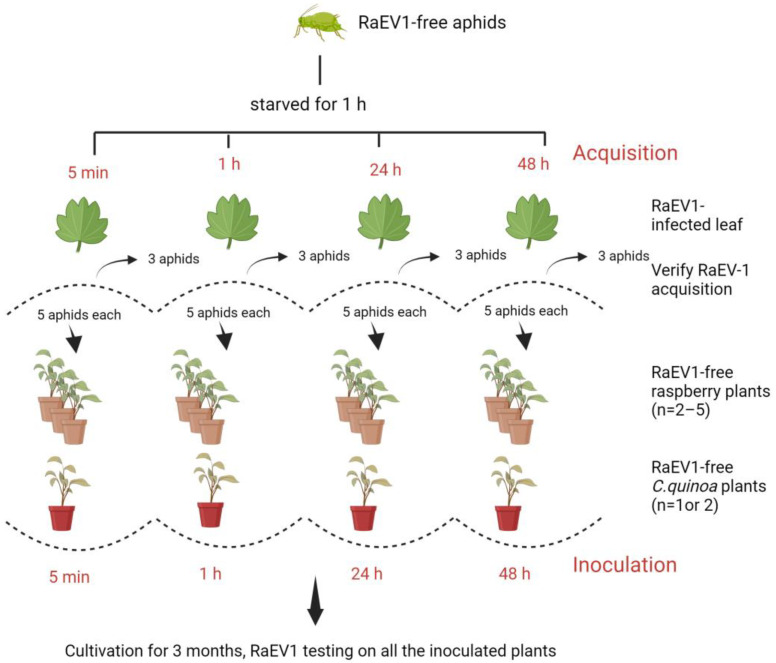
Schematic of the assay for aphid-assisted transmission of raspberry enamovirus 1 (RaEV1) to raspberry and *Chenopodium quinoa* plants. Created with BioRender.com (agreement number TI2649MVLL).

The aphids were allowed to feed on RaEV1-infected shoots for 5 min, 1 h, 24 h, and 48 h (28 aphids in each acquisition group). After the acquisition period, a batch of five aphids was placed on the upper surface of the RaEV1-free test plants (*C. quinoa* and *R. idaeus*). Following the transfer, the aphids were left for four inoculation periods (5 min, 1 h, 24 h, and 48 h; Figure 1).

The experimental treatments of different aphids and the acquisition and inoculation times are listed in Table 1. To verify whether aphids had acquired the virus, three aphids were collected after each acquisition treatment and tested with RT-PCR for RaEV1. Effective RNA extraction was confirmed by amplifying the COI gene, and the detection of plant material was assessed through NADH gene amplification (Table S1).

### 2.9. Data Analysis

All sequence data were analyzed using Geneious Prime^®^ 2023.0.4 (Biomatters, Auckland, New Zealand) and CLC Genomics Workbench 9.5.1 (Qiagen). Open reading frames (ORFs) were identified using ORFfinder, BLASTp annotation, and comparative alignments with the related viral sequences. The HTS data were deposited in the NCBI archive.

### 2.10. Phylogenetic Analyses

Multiple protein alignments were carried out in Geneious software using the MAFFT algorithm. For the phylogenetic analysis, alignments with all gap positions removed were processed for tree reconstruction using PhyML 3.1 [18], implementing the Whelan and Goldman (WAG) model. Statistical tests for branch support were performed using the approximate likelihood-ratio test [19] with minimum Shimodaira–Hasegawa/Chi2-based support values. The phylogenetic trees thereby obtained were visualized using the iTOL v3 tool [20] and rearranged in Affinity Publisher 1.10.6 (Serif Europe Ltd., Nottingham, UK).

## 3. Results and Discussion

### 3.1. High-Throughput Sequencing

Three plants were screened for viruses using the HTS approach. A total of 119–214 million paired reads were acquired per sample, and two of them were deposited in the NCBI SRA under BioProjectID PRJNA1028176. After undergoing quality control and adapter trimming, these reads were subjected to de novo assembly. Subsequently, the resulting contigs were utilized for a viral search against a custom local BLAST database of viral proteins, which was constructed on 11 October 2023. None of the three datasets were free of viral sequences (Table 2). Several sequences related to enamoviruses were detected.

After conducting nucleotide comparisons within each sample, our analysis led us to the conclusion that these three sequences indeed correspond to isolates of a novel viral species, as their genetic diversity did not surpass 12%. In addition to the enamovirus-like sequences, the ALOJ-12 and ALOJ-13 samples contained signatures of other raspberry viruses (Table 2). Generally, the number of viral reads was rather low and, in total, did not exceed 0.75%.

**Figure 2:**
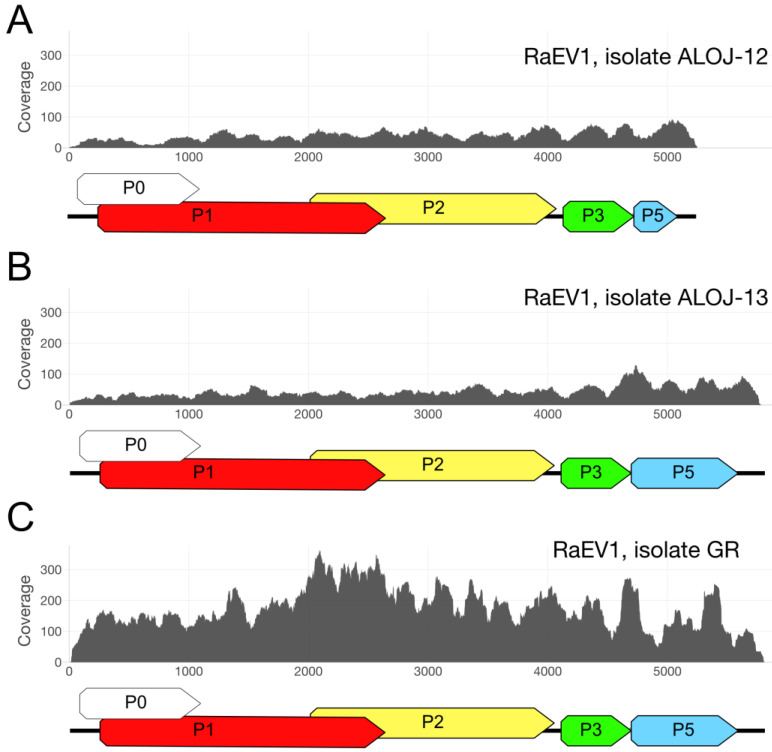
High-throughput sequencing coverage of three enamovirus sequences derived from raspberry isolates: (**A**) ALOJ-12, (**B**) ALOJ-13, and (**C**) GR. The *X*-axis represents nucleotide positions, while the *Y*-axis indicates coverage values. A predicted genomic scheme is depicted below each coverage plot. For an in-depth genome description, please refer to the next section.

Analyzing the number of reads and the sequencing coverage profile, we found that the dsRNA library (Figure 2C) had half as many enamovirus-like reads, and the sequencing coverage profile differed from that prepared from the total RNA (Figure 2A,B).

For the other samples, the coverage slowly decreased from the 3′ to the 5′ terminus, and there were pronounced peaks in the middle of the GR sequence. Importantly, throughout the sequences, the coverage profiles remained consistent without any noticeable irregularities.

### 3.2. Genome

**Figure 3:**
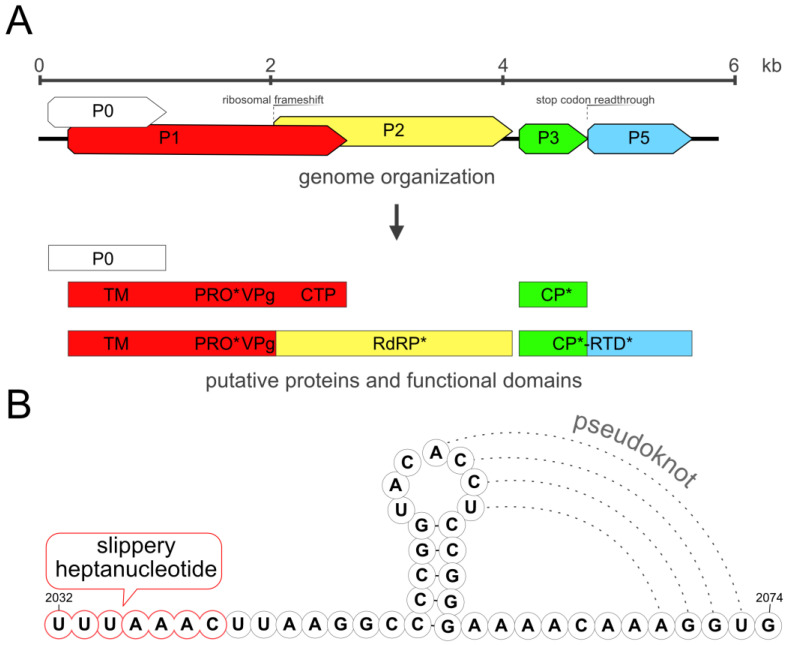
(**A**) Genome organization of raspberry enamovirus 1 GR isolate. Domain abbreviations: TM—transmembrane domain, PRO—protease domain; VPg—virus protein genome-linked; CTP—C-terminal domain; RdRP—RNA-dependent RNA polymerase; CP—coat protein; RTD—readthrough domain. (**B**) The ribosomal slippery motif and downstream secondary structures. *—Functional domains marked with an asterisk were identified using the Motif Scan web service (https://myhits.sib.swiss, accessed on 12 September 2023), while others were predicted based on the description of enamoviruses.

The genomic sequence of the RaEV1 GR isolate was confirmed through Sanger sequencing and RACE procedures and was deposited in the GenBank under accession number OR683427. The complete genome spanned 5824 nucleotides (nts). Five putative open reading frames were predicted, and their numbering conformed to the typical conventions observed in enamoviruses (Figure 3A).

ORF0 (from 97 to 1089 nts) encodes a putative silencing suppression protein (P0) and partially overlaps with ORF1. ORF1 (from 266 to 2035 nts) encodes a transmembrane domain, peptidase, virus protein genome-linked, and, if a ribosomal frameshift occurs, an RNA polymerase domain; otherwise, it encodes a C-terminal domain (Figure 3A). A fusion protein, known as the replicase polyprotein, is generated through the −1 ribosomal frameshift mechanism, involving both ORF1 (from 266 to 2035 nts) and ORF2 (from 2035 to 4080 nts). There is a UUUAAAC motif known to facilitate ribosomal slippage shared with potato leafroll virus (PLRV) and several other enamoviruses. Two secondary structures, a stem and a pseudoknot, were predicted downstream of the motif (Figure 3B).

ORF3 (from 4145 to 4726 nts) encodes a putative coat protein. A fusion protein, referred to as CP-readthrough domain protein (CP-RTP), is synthesized through a read-through mechanism of the stop codon of ORF3 (from 4145 to 5626 nts) and spans ORF5. The CP-RTD protein is a multifunctional protein that participates in various stages of the viral life cycle, including systemic movement within the plant, phloem loading, the survival of virions in aphid vectors, and tissue tropism [7,21].

### 3.3. Phylogeny

**Figure 4:**
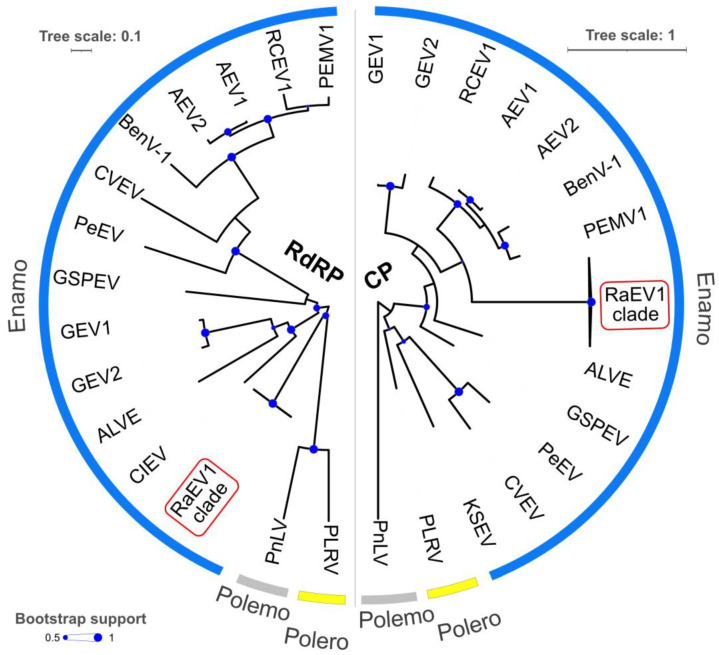
Phylogenetic trees based on the alignment of the deduced amino acid sequences of the polymerase (RdRP) and coat proteins (CPs) of raspberry enamovirus 1 (RaEV1, accession number OR683414-27) and members of the *Enamovirus* (blue), *Polerovirus* (yellow)*,* and *Polemovirus* (grey) genera. The viruses used to construct the tree, along with their accession numbers, are as follows: alfalfa enamovirus 1 (AEV1)—KU297983; alfalfa enamovirus 2 (AEV2)—KY985463; arracacha latent virus E (ALVE)—MF136435; bean enamovirus 1 (BenV-1)—MZ361924; Celmisia lyallii enamovirus (CIEV)—BK059370; citrus vein enation virus (CVEV)—ON494593; grapevine enamovirus 1 (GEV1)—MT536978; grapevine enamovirus 2 (GEV2)—OR066156; green Sichuan pepper enamovirus (GSPEV)—MH323436; Kummerowia striatad enamovirus (KSEV)—MN814310; pea enation mosaic virus 1 (PEMV1); NC_003629 pepper enamovirus (PeEV)—MG470803; potato leafroll virus (PLRV)—D00530; poinsettia latent virus (PnLV)—AJ867490; and red clover enamovirus 1 (RCEV1)—MG596229.

To investigate the relationships of RaEV1 with other enamoviruses, we used both the polymerase and coat proteins of fourteen RaEV1 isolates for analyses (Figure 4). To depict the phylogenetic relationships among the different virus species more clearly, the branches containing RaEV1 isolates were collapsed. A detailed version of the phylogenetic trees is presented in Figure S1. The reconstruction of the phylogeny revealed that, for the polymerase, the RaEV1 isolates formed a separate clade separated from potato leafroll virus and poinsettia latent virus (Figure 4, RdRP). However, for the coat protein, the RaEV1 clade was clustered within the enamovirus group.

### 3.4. Shared Protein Identity with Other Enamoviruses

**Figure 5:**
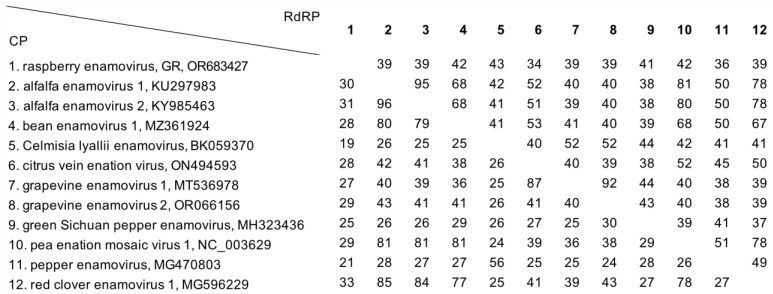
Overview of shared pairwise identities (%) of RaEV1 and other enamoviruses in CP (lower triangle) and RdRP (upper triangle).

Pairwise comparisons of amino acid identities between RaEV1 and both putative and recognized members of the *Enamovirus* genus revealed limited conservation (Figure 5).

The current species demarcation criteria within the genus stipulate at least a 10% difference in amino acid residues for any given protein [9]. With RaEV1 sharing no more than 33% of amino acid residues in CP and 43% in RdRP, it can be considered a distinct species within the *Enamovirus* genus.

### 3.5. Isolate Diversity

**Figure 6:**
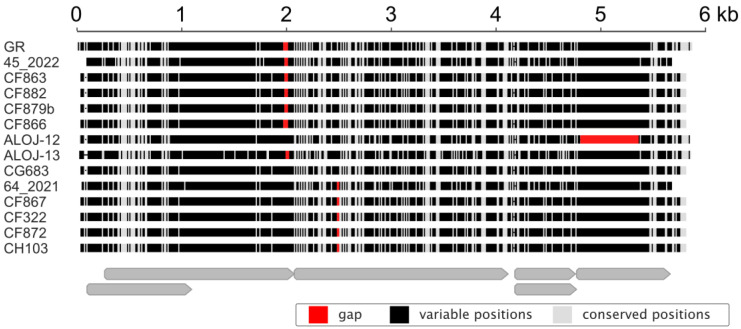
Overview of the multiple alignment of fourteen isolates of RaEV1 with highlighted conserved, variable, and indel (gap) regions.

To estimate the genetic variability of RaEV1, we sequenced eleven isolates using the Sanger approach. The alignment of nearly complete genome sequences from fourteen RaEV1 isolates revealed pairwise identities ranging from 85% to 96%, with the majority being found in the 5′ part of the genomes (Figure 6). Except for ALOJ-12, which had 85–88% nt identities with other isolates, the isolates exhibited over 93% conservation.

Notably, nearly identical isolates (99.8%) were detected in two different cultivars from two sites situated roughly 60 km apart: A807 (GenBank accession number OR683421, raspberry cv. Bulharský rubín) and A709 (OR683415, raspberry cv. Canby). Comparative analyses of the CP and RdRP across the RaEV1 isolates revealed high conservation levels. Specifically, the isolates exhibited more than 95% and 98% amino acid identities in RdRP and CP, respectively.

Furthermore, several indel events were observed in the aligned genomic sequences (Figure 6). Upstream of the slippery motif at approximately 2 kb, indels ranging from 24 to 33 nts were observed, and 3 nt long indels were observed approximately 500 nts downstream (Figure 6). Meanwhile, the P5 ORF of the ALOJ-12 isolate showed a substantial deletion relative to those of the other isolates. Interestingly, the deletion did not disrupt the reading frame, but the predicted fusion CP-RTD protein was truncated by 186 amino acids. The deletion might have a large impact on the viral life cycle, as the CP-RTD protein has not only a structural function but, for example, in cucurbit aphid-borne yellows virus (the *Polerovirus* genus), also has a soluble form that contributes to phloem limitation and in planta movement [22]. Consequently, a mutant with aborted CP-RTD synthesis accumulates very poorly in upper noninoculated leaves [22]. For PLRV, a polerovirus, it was shown that the readthrough P5 part, the RTD, is responsible for aphid transmission. It was shown that the RTD protein is proteolytically cleaved [10] and therefore it is probable that the ALOJ-12 isolate’s RTD part is missing a cleavage site. However, it was proposed that readthrough events in members of the *Enamovirus* genus are influenced by RNA–RNA interactions between secondary structures from two regions downstream of the readthrough termination codon, and the distance between these regions may vary among different viruses. As a result, the possibility that, despite the deletion, the readthrough part remained intact in the ALOJ-12 isolate could not be excluded. Additionally, the absence of this sequence significantly affects the pairwise identities between isolate ALOJ-12 and the other samples. When this region is omitted, the identities fall within a range of from 93.4% to 97.7%.

### 3.6. Prevalence

The alignment of fourteen distinct RaEV1 isolates allowed for moderate insight into the virus variability and subsequent design of the detection primers. We used several pairs of detection primers for the virus (Table S1).

**Figure 7:**
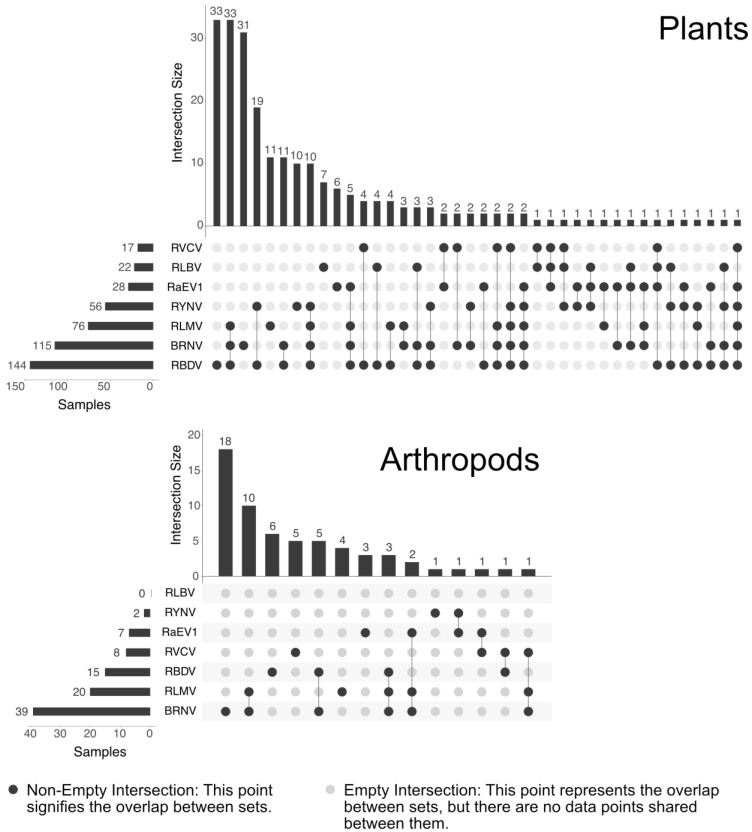
UpSet plots displaying the virus infection compositions of the raspberry and arthropod samples tested for RaEV1 and other prevalent raspberry viruses. Note: 62 plant and 107 invertebrate samples tested negative and were excluded from these representations.

In total, 289 plant samples and 168 invertebrate samples were collected and tested. Of these, 10.4% of the plant samples and 0.4% of the invertebrate samples tested positive for RaEV1, with most cases showing mixed virus infections (Figure 7, Table S2).

From the detection results, it was notable that BRNV, RBDV, and RLMV were found frequently together (Figure 7A). At almost the same rate, single infections with BRNV and RBDV were documented. Notably, although BRNV and RLMV are transmitted by aphid vectors, RBDV is pollen- and seed- transmitted. The frequently found association of BRNV and RDBV together with raspberry yellow net virus (RYNV) is believed to be responsible for raspberry mosaic disease [5]. In our analysis of the plant samples, RBDV emerged as the most prevalent virus, irrespective of whether it was in a mixed infection or present alone. Notably, the prevalence of RaEV1 was higher than that of RLBV and RVCV.

In the analysis of the invertebrate samples, BRNV was predominantly detected, either individually or in conjunction with RLMV. RLBV, being mite-vectored, was not detected in any of the samples, as expected. Notably, three of the seven RaEV1-positive samples contained only this virus.

**Figure 8:**
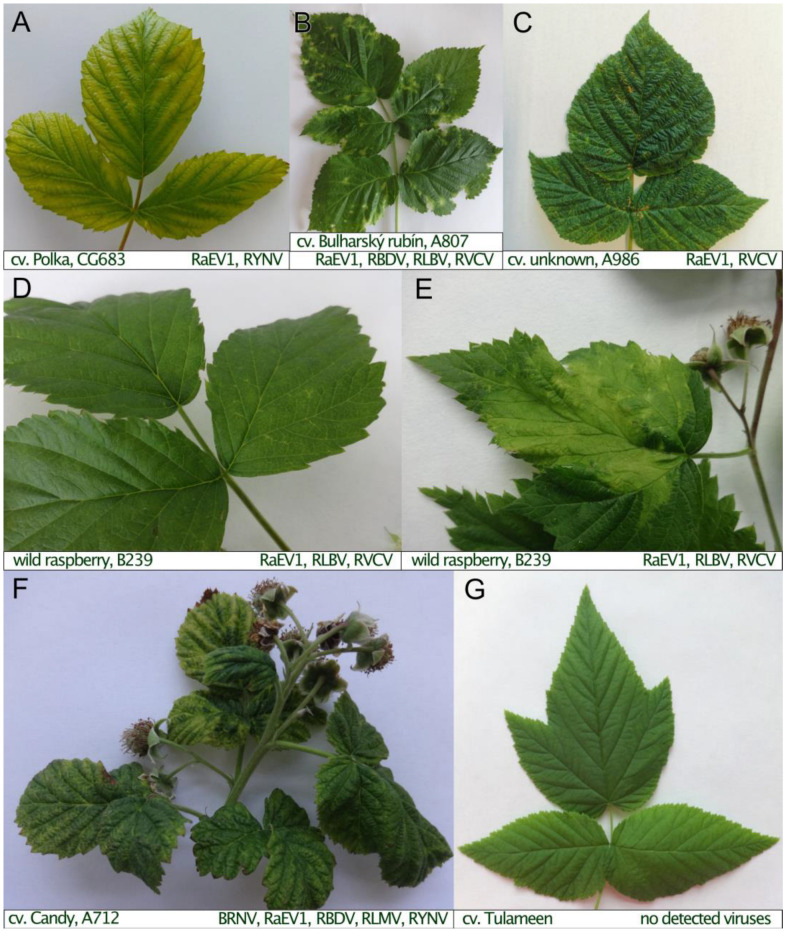
Symptoms of RaEV1-positive raspberry plants showing (**A**) yellowing, (**B**) diffuse lesions, leaf blotching, isolate NB1, (**C**) leaf curl, necrosis, and slight vein clearing, (**D**) slight vein clearing, (**E**) leaf blotching on different leaves of isolate B239 (HL6), and (**F**) mosaic, leaf curl, and necrosis. (**G**) Leaf of virus-negative tested raspberry shown as control. BRNV: black raspberry necrosis; RaEV1: raspberry enamovirus 1; RBDV: raspberry bushy dwarf; RLBV: raspberry leaf blotch virus; RLMV: raspberry leaf mottle virus; RVCV: raspberry vein chlorosis virus; RYNV: Rubus yellow net virus.

While virus screening was conducted on various raspberry samples, the visual state was also recorded. Interestingly, the only single-RaEV1 infection found in the GR plant did not cause any obvious visual symptoms. Similarly, no symptoms resembling those of viral infection were observed in the HL2 plant (CZ) or in the nine plants from Norway in which RaEV1 was detected in mixed infections with other viruses (Table S2). However, various virus-like symptoms were observed in other plants (Table S2, Figure 8).

Considering that plant viruses often infiltrate and establish themselves differently across various plant components, we questioned whether RaEV1 dwells in only specific plant organs or whether it is omnipresent. For two plants, HL2 and HL6, we confirmed the presence of RaEV1 in the following parts: the roots, stems, leaves, buds, flowers, green fruits, red fruits, and receptacles.

The positive-tested samples included commonly used cultivars in raspberry production, such as Polka, Glen Mor, and Glen Ample. Notably, RaEV1 was detected in both cultivated and wild raspberries, suggesting that wild plants may serve as virus reservoirs. Wild raspberries were collected from different regions both in the Czech Republic and in Norway (Table S2), indicating that the virus infection is not solely localized.

While sampling raspberries, insect samples were collected and tested in parallel (Table S2). A few samples of *Am. rubi idaei*, *A. idaei*, *Psallus wagneri* (Miridae), and *Macropsis fuscula* (Cicadellidae) colonizing RaEV1-positive plants tested positive for the virus (Table S2). While aphids are widely recognized as vectors of plant viruses, members of the Miridae family are predominantly known as agricultural pests and have not yet been confirmed as vectors of plant viruses. However, the rubus leafhopper, *M. fuscula*, is primarily identified as a vector for the ‘*Candidatus* Phytoplasma rubi’ [23], and the detection of RaEV1 should be addressed in future studies.

Interestingly, while the *A. idaei* larvae collected from sample 56/2022 were virus-negative, the aphid adults were virus-positive. We also included other nonaphid species in the screening to widen the possibility of detecting potential vectors, although none of them were found to be RaEV1-positive. It is also worth noting that we found virus-positive *Am. rubi idaei* and *A. idaei* aphids on plants that tested negative for the virus. Future tests on these plants are essential to ensure that we did not observe a momentary introduction of the infection.

### 3.7. Transmission Assays

The presence of RaEV1 in *Am. rubi idaei* and *A. idaei* has led us to hypothesize that these species may act as virus vectors. *M. persicae* can act as a vector for over 100 plant viruses [24] and was found to colonize and feed on raspberry plants in a preliminary experiment. Therefore, virus transmission by *Am. rubi idaei, A. idaei*, and *M. persicae* was tested.

**Figure 9:**
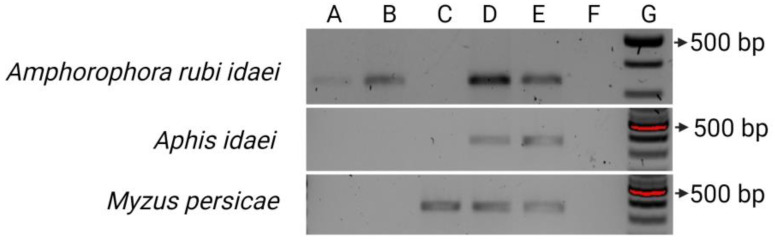
Gel picture of RT-PCR detection results for RaEV1 in collected aphids after different acquisition periods. From A to D: aphids collected after 5 min, 1 h, 24 h, and 48 h acquisition times, respectively; E: RaEV1 positive control; F: RaEV1 negative control; G: 100 bp ladder. The size of the targeted band for RaEV1 was 347 bp. Created with BioRender.com (agreement number SO2649NMPJ).

The aphid start cultures were tested for RaEV1 before transmission and proved to be RaEV1-free. Different acquisition times ranging from 5 min to 48 h were tested by feeding the aphids RaEV1-infected raspberry leaves. RaEV1 was acquired by *Am. rubi idaei* after 5 min, 1 h, and 48 h of acquisition (Figure 9), but 24 h of acquisition could not be assessed due to failed RNA extraction from collected aphids (the aphid internal control was negative).

RaEV1 was acquired by *A. idaei* after 48 h of acquisition (Figure 9). *M. persicae* acquired RaEV1 after 24 h and 48 h, but not within the other shorter acquisition times tested (Figure 9). All RaEV1-positive aphids gave negative results for the plant internal Nad control to exclude the possibility of plant debris inside the aphids. These results proved that RaEV1 was acquired successfully in the aphid body.

All inoculated plants were tested for RaEV1 via RT-PCR three months after inoculation. None of the tested plants were positive for RaEV1, indicating that none of the three aphid species acted as a vector for the transmission of RaEV1 to either raspberry or *C. quinoa* plants under the experimental conditions. We cannot exclude the possibility of viral isolate or species incompatibility. Enamoviruses have an intimate relationship with their vectors, and the transmission process is a series of highly specific events in which viral particles must overcome several barriers [8,25]. Vector specificity for viral isolates results from the ability of virions to penetrate the accessory salivary glands of vector aphids [7]. However, viral particles can still persist in the hemocoels of the non-vector aphids [7,8], which can lead to positive viral detection after certain acquisition periods, even when transmission is not feasible.

## 4. Conclusions

With a growing number of plant samples undergoing high-throughput analyses, we are seeing increasing numbers of novel viral species. In this study, we reported a novel virus infecting raspberries. Based on molecular and phylogenetic evidence, it belongs to a novel species within the *Enamovirus* genus and we propose to give the species the name ‘raspberry enamovirus 1′. We showed that the virus was present in wild and cultivated raspberries as a single infection or in combination with other raspberry viruses. As an enamovirus, RaEV1 is likely transmitted by an aphid vector. Unfortunately, experiments with *Am. Rubi idaei, A. idaei*, and *M. persicae* aphids did not result in its successful transmission. However, it cannot be excluded that the RaEV1 isolates and aphid biotypes from the current study did not match.

Our study sheds light on the existence of this novel raspberry-infecting virus and raises intriguing questions about its transmission dynamics and potential interactions with host plants and vectors, which will require additional research to fully understand.

## Figures and Tables

**Table 1:** Transmission experiment with various aphids and acquisition and inoculation periods for RaEV1; *nt*—not tested; min—minutes; h—hour/hours.

Aphid Species	Treatment ID	Acquisition Period	Inoculation Period	No. and Type of Inoculated Plants
*Aphis idaei*	1	5 min	*nt*	Five individual virus-free raspberry plants in each treatment
	2	1 h	1 h	
	3	24 h	24 h	
	4	48 h	48 h	
*Amphorophora rubi idaei*	5	5 min	5 min	Two individual virus-free raspberry plants and two *Chenopodium quinoa* seedling plants in each treatment
	6	1 h	1 h	
	7	24 h	24 h	
	8	48 h	48 h	
*Myzus persicae*	9	5 min	5 min	Two individual virus-free raspberry plants and one *C. quinoa* seedling plant in each treatment
	10	1 h	1 h	
	11	24 h	24 h	
	12	48 h	48 h	

**Table 2:** Counts of reads and their corresponding percentages from trimmed reads aligned to viral contigs.

Virus	Sample		
	GR	ALOJ-12	ALOJ-13
Raspberry enamovirus 1	6883 (0.0032%)	1482 (0.0019%)	1759 (0.002%)
Raspberry leaf blotch virus	0	362,327 (0.45864%)	0
Black raspberry necrosis virus	0	213,144 (0.26980%)	70 (0.00008%)

## Data Availability

The data presented in this study were deposited in the NCBI SRA storage repository under BioProjectID PRJNA1028176 and in the GenBank database under accession numbers OR683414-OR683427, or they are available upon request.

## Supplementary Materials

The following supporting information can be downloaded at https://www.mdpi.com/article/10.3390/v15122281/s1, Figure S1: Phylogenetic trees; Table S1: List of primers used in the study; Table S2: Virus detection in plant and invertebrate samples.
