# Supplementary material for: Molecular Characterization of a Novel Enamovirus Infecting Raspberry
Source: Viruses. 2023 Nov 21;15(12):2281. doi: 10.3390/v15122281 (PMC10747458; doi:10.3390/v15122281)
Supplement: Supplementary file 1 [file viruses-15-02281-s001.zip › Supplementary Figure S1.pdf]

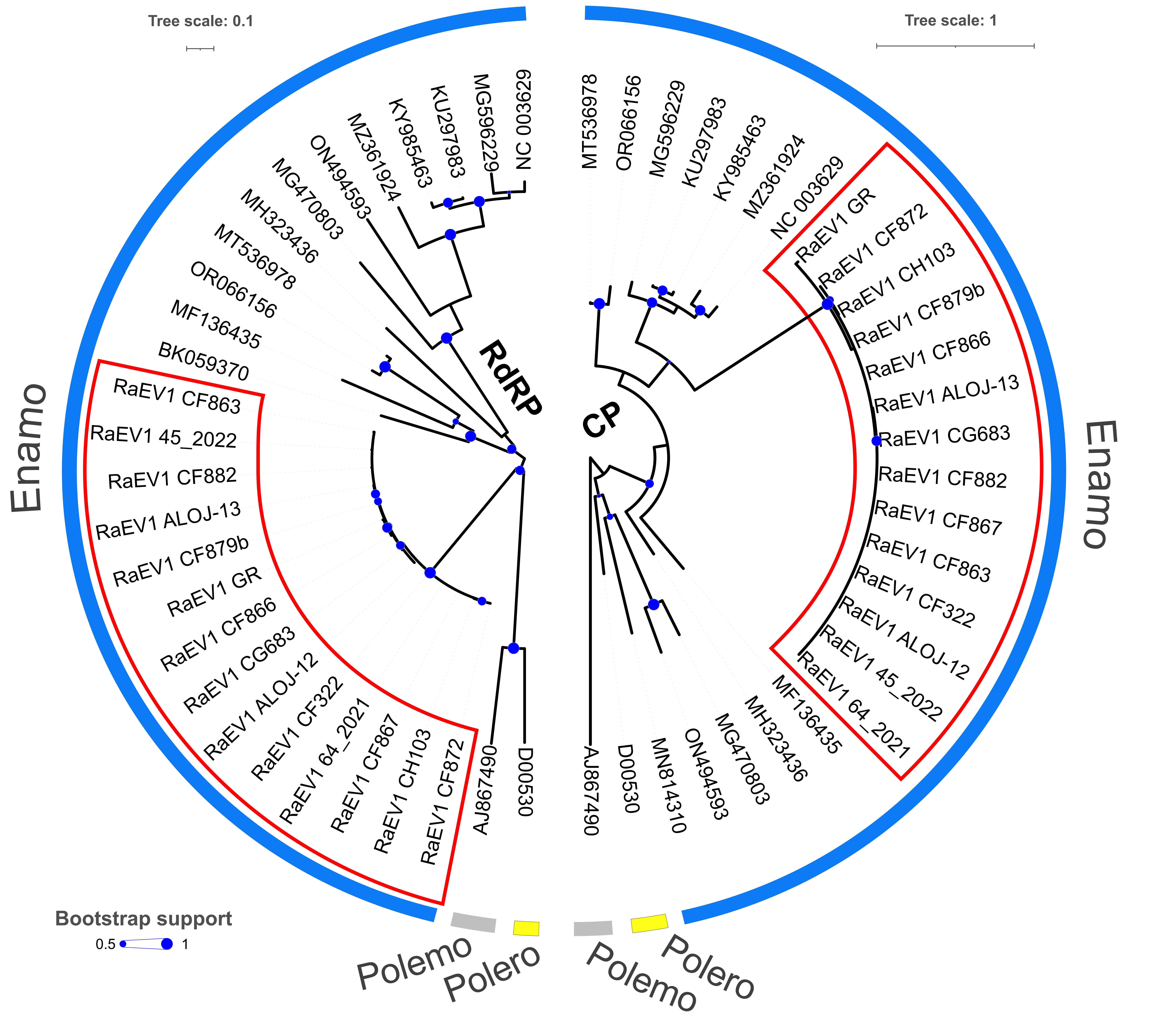

Figure S1 Phylogenetic trees based on the alignment of the deduced amino acid sequences of the polymerase (RdRP) and coat (CP) proteins of raspberry enamovirus 1 isolates (RaEV1, accession numbers OR683414-27), and members of the Enamovirus (blue), Polerovirus (yellow), and Polemovirus (grey) genera. The viruses used to construct the tree, along with their accession numbers, are as follows – alfalfa enamovirus 1 (AEV1) – KU297983, alfalfa enamovirus 2 (AEV2) – KY985463, arracacha latent virus E (ALVE) – MF136435, bean enamovirus 1 (BenV-1) – MZ361924, Celmisia lyallii enamovirus (CIEV) – BK059370, citrus vein enation virus (CVEV) – ON494593, grapevine enamovirus 1 (GEV1) – MT536978, grapevine enamovirus 2 (GEV2) – OR066156, green Sichuan pepper enamovirus (GSPEV) – MH323436, Kummerowia striatad enamovirus (KSEV) – MN814310, pea enation mosaic virus 1 (PEMV1) , NC_003629 pepper enamovirus (PeEV) – MG470803, potato leafroll virus (PLRV) – D00530, poinsettia latent virus (PnLV) – AJ867490, and red clover enamovirus 1 (RCEV1) – MG596229.
